# Development of Cerebellar Neurons and Glias Revealed by in Utero Electroporation: Golgi-Like Labeling of Cerebellar Neurons and Glias

**DOI:** 10.1371/journal.pone.0070091

**Published:** 2013-07-23

**Authors:** Yoshiaki Kita, Koichi Kawakami, Yoshiko Takahashi, Fujio Murakami

**Affiliations:** 1 Graduate School of Frontier Biosciences, Osaka University, Suita, Osaka, Japan; 2 Division of Molecular and Developmental Biology, National Institute of Genetics, Mishima, Shizuoka, Japan; 3 Department of Genetics, The Graduate University for Advanced Studies (SOKENDAI), Mishima, Shizuoka, Japan; 4 Department of Zoology, Graduate School of Science, Kyoto University, Kyoto, Kyoto, Japan; Institut de la Vision, France

## Abstract

Cerebellar cortical functions rely on precisely arranged cytoarchitectures composed of several distinct types of neurons and glias. Studies have indicated that cerebellar excitatory and inhibitory neurons have distinct spatial origins, the upper rhombic lip (uRL) and ventricular zone (VZ), respectively, and that different types of neurons have different birthdates. However, the spatiotemporal relationship between uRL/VZ progenitors and their final phenotype remains poorly understood due to technical limitations. To address this issue, we performed in utero electroporation (IUE) of fluorescent protein plasmids using mouse embryos to label uRL/VZ progenitors at specific developmental stages, and observed labeled cells at maturity. To overcome any potential dilution of the plasmids caused by progenitor division, we also utilized constructs that enable permanent labeling of cells. Cerebellar neurons and glias were labeled in a Golgi-like manner enabling ready identification of labeled cells. Five types of cerebellar neurons, namely Purkinje, Golgi, Lugaro and unipolar brush cells, large-diameter deep nuclei (DN) neurons, and DN astrocytes were labeled by conventional plasmids, whereas plasmids that enable permanent labeling additionally labeled stellate, basket, and granule cells as well as three types of glias. IUE allows us to label uRL/VZ progenitors at different developmental stages. We found that the five types of neurons and DN astrocytes were labeled in an IUE stage-dependent manner, while stellate, basket, granule cells and three types of glias were labeled regardless of the IUE stage. Thus, the results indicate the IUE is an efficient method to track the development of cerebellar cells from uRL/VZ progenitors facing the ventricular lumen. They also indicate that while the generation of the five types of neurons by uRL/VZ progenitors is regulated in a time-dependent manner, the progenitor pool retains multipotency throughout embryonic development.

## Introduction

The cerebellum is composed of distinct types of neurons that have unique morphological features and synaptic connectivities [1]. The precise order of the cellular arrangements and neuronal circuitries are thought to play important roles on essential functions like motor control and learning (reviewed in [2]). Thus, much attention has been given to how the neuronal architecture of the cerebellum is constructed.

The development of the cerebellum has been studied extensively [3], [4], and the generation of cerebellar neurons has been shown to be both temporally and spatially regulated. Deep nuclei (DN) neurons, Purkinje cells, inhibitory interneurons and granule cells are generated sequentially [5]–[8], although the final division of granule cells occurs in the external granular layer (EGL) [9]–[11] and that of Golgi cells, stellate cells and basket cells in the white matter (WM) [12], [13]. Recent genetic fate mapping (GFM) studies showed that excitatory neurons like large-diameter DN neurons and granule cells and inhibitory neurons like Purkinje, Golgi, stellate, and basket cells respectively originate from spatially distinct regions, the upper rhombic lip (uRL) [14]–[18] and the adjacent ventricular zone (VZ) [8], [19].

Although these studies have made a great contribution to our understanding of the spatial origin of cerebellar cells, they do have their limitations. For example, transcription factor expression also occurred in the brainstem [14], [15], [19]–[21], suggesting contributions from this region cannot be dismissed. Even within the cerebellar anlage, transcription factor expressions are not necessarily confined to the progenitors facing the lumen of the IVth ventricle (e.g., [20]), making it difficult to examine the relationship between these cells and their progeny. Moreover, the final division of some cerebellar neurons occurs in the EGL [9]–[11] and the WM [12], [13], obfuscating the spatiotemporal relationship between the final phenotype of the cerebellar cells and the corresponding progenitor origins at specific developmental stages.

Gene transfer by in utero electroporation (IUE) is a method that can provide information that has not been accessible by GFM, because this method allows specific labeling of progenitors facing the IVth ventricular lumen of the cerebellar anlage and at specific developmental stages. Here we applied this method to study cerebellar cell development and used three different types of plasmid: conventional plasmids encoding for GFP or mCherry; a set of plasmids that allows for the integration of genes with the aid of a transposase [22]; and a Cre-recombinase expression plasmid combined with a reporter mouse line, Ai9 [23]. By combining these methods, we show here that all types of cerebellar neurons and glias originate from progenitors facing the lumen of the cerebellar anlage. We also show that Purkinje cells, Golgi cells, unipolar brush cells (UBCs), large-diameter DN neurons, and DN astrocytes almost directly arise from labeled progenitors at respective specific developmental stages, whereas precursors of granule cells, stellate/basket cells, Bergmann glia, oligodendrocytes, and cortical astrocytes stay in the uRL/VZ and continue proliferation over a protracted period of embryonic development.

## Materials and Methods

### Animals

All procedures were performed in accordance with the Osaka University Guidelines for the Welfare and Use of Laboratory Animals and approved by the Committee on Animal Care of the Graduate School of Frontier Biosciences of Osaka University (Permit Number: FBS-12-004). Timed-pregnant ICR mice (Nihon-SLC) and mice homozygous or heterozygous for the Ai9 allele [23] on C57BL/B6J or ICR backgrounds were used for the lineage tracing of uRL/VZ progenitors. Noon of the day of the vaginal plug detection was termed embryonic day (E) 0.5, and E19.5 was defined as postnatal day (P) 0.

### Expression Vectors

pCAGGS-EGFP [24], [25], pCAGGS-mCherry [26], pT2K-CAGGS-EGFP [27], and pCAGGS-T2TP [28], [29] vectors have been described previously. pT2K-CAGGS-EGFP is a Tol2 transposon-flanked EGFP and pCAGGS-T2TP codes for Tol2 transposase. Upon the co-introduction of pT2K-CAGGS-EGFP and pCAGGS-T2TP into a cell, the resulting transposon construct was excised from the plasmid and integrated into the host genome [22]. IUE experiments have indicated that Tol2 transposase stably integrates the Tol2-flanked transgene into the genome of proliferative neural progenitors [30]. The mixture of pCAGGS-T2TP and pT2K-CAGGS-EGFP is referred to as Tol2-GFP hereafter. pCAGGS-Cre [31] and pCALNL5-EGFP [32] were provided by Dr. J. Miyazaki and Dr. K. Yamauchi, respectively. pCALNL5-EGFP encodes EGFP and expression was induced by Cre-mediated removal of a stop cassette flanked by loxP sites [33].

### In Utero Electroporation (IUE)

IUE was performed as described [34], [35] with some modifications. E10.5–E15.5 embryos were used because there is a minimal mitotic activity before E10.5 in the cerebellar primordium [9] and because mitotic activity of the “primitive ependymal zone” is reduced between E13.5–E15.5 [5]. Electric pulses (30 V at E10.5, 40 V at E11.5, 50–60V at E12.5, E13.5, E14.5 and E15.5; five times in 950 ms intervals) were then delivered with forceps-shaped electrodes (CUY650P2 or CUY650P3, Unique Medical Imada) connected to an electroporator (CUY21, Nepa Gene). Plasmids were dissolved in phosphate-buffered saline (PBS) (0.1 M, pH 7.4) and used at the following final concentrations: pCALNL5-EGFP (1 µg/µl), pCAGGS-Cre (2× 10^−3^ µg/µl) and pCAGGS-mCherry (1 µg/µl) for sparse labeling of cells; pCAGGS-T2TP (2 µg/µl), pT2K-CAGGS-EGFP (1 µg/µl) and pCAGGS-mCherry (1 µg/µl) for the transposon-mediated gene transfer; and pCAGGS-Cre (1 µg/µl) and pCAGGS-EGFP (1 µg/µl) for lineage tracing using Ai9 reporter mice [23].

### Tissue Processing

Embryos were decapitated and postnatal mice were perfused transcardially. Brains were dissected and processed as previously described [26], except that sections were cut at 40 µm. For the analysis of labeled cells in postnatal brains, every eighth to tenth sagittal or coronal section was examined.

### Identification of Cell Types by Molecular Markers

Neurons and glias were first identified by their morphological features, and these conclusions confirmed by measuring the expressions of different molecular markers.

### Immunohistochemistry

Sections were immunostained as described previously [26] except that 0.5% Triton X-100 was used. The primary antibodies used were as follows: rat monoclonal anti-GFP antibody (clone GF090R, 1∶1000; 04404-84; Nacalai Tesque), rabbit anti-DsRed antibody (1∶300; 632496; Clontech), mouse monoclonal anti-calbindin D28k antibody (clone 300, 1∶2000; Swant), mouse monoclonal anti-parvalbumin antibody (clone 235, 1∶1000; Swant), rabbit anti-neurogranin antibody (1∶200; AB5620; Millipore), rabbit anti-GFAP antibody (1∶1000; G9269-.2 ML; Sigma), mouse monoclonal anti-MBP antibody (clone 26, 1∶500; MBP384; Millipore), mouse monoclonal anti-PCNA antibody (clone PC 10, 1∶1000; p8825; Sigma), and rabbit anti-nestin antibody (1∶1000; gifted by Dr. Y. Arimatsu). The secondary antibodies used were Alexa Fluor 488-conjugated anti-rat IgG (1∶400), Alexa Fluor 488-conjugated anti-mouse IgG (1∶400), Alexa Fluor 594-conjugated anti-rabbit IgG (1∶500), Alexa Fluor 647-conjugated anti-rabbit IgG (1∶500), and Alexa Fluor 647-conjugated anti-mouse IgG (1∶500) and purchased from Invitrogen. For PCNA immunohistochemistry, sections were treated with citrate buffer (0.01 M citric acid, pH 6.0) at 98–99°C for 20 min to retrieve antigens before incubation with the primary antibody.

### BrdU Labeling

To examine the mitotic activity of the cells leaving the ventricular surface, electroporation of GFP was performed at E13.5 and on the next day the dam was intraperitoneally injected with 5-bromo-2-deoxyuridine (BrdU) (Sigma; 10 mg/ml in PBS) at 50 mg/kg body weight at 9∶30 A.M., 12∶30 p.m., and 15∶30 p.m. Brains were dissected out 30 min after the last injection. For double immunostaining for GFP protein and incorporated BrdU, 25 µm-thick sagittal sections of the cerebellar primordium were prepared. These sections were stained with chicken anti-GFP antibody (1∶1500; ab13970; Abcam) and Alexa Fluor 488-conjugated anti-chicken IgG (1∶500, Jackson ImmunoResearch), followed by incubation in 2N HCl for 30 minutes at 37°C and borate buffer (0.1 M boric acid, pH 9.0) for 10 minutes at room temperature. They were then rinsed in 0.5% PBST and reacted with a rat monoclonal anti-BrdU antibody (BU1/75 (ICR1), 1∶200, Abcam) overnight at 4°C. After extensive washing with 0.5% PBST and PBS, the sections were incubated with an Alexa Fluor 594-conjugated anti-rat IgG antibody (1∶400, Invitrogen) for 2 hours at room temperature.

### Image Processing

Images were captured using a CCD camera (AxioCam; Zeiss) attached to an epifluorescence microscope (BX60 or BX61; Olympus) or by a confocal microscope (TCS SP2, Leica). Stacks of confocal images were taken at 0.6–1.2 µm intervals with either a 40X (N.A., 0.85) or a 100X lens (N.A., 1.4). Maximum projection images were created using confocal software (Ver. 2.61, Leica) or Image J software (Ver. 1.46G, National Institutes of Health). Captured images were processed to adjust the contrast using Photoshop 7.0 or Photoshop Elements 5.0 (Adobe Systems).

### Quantification of the Diameter of DN Neurons

Images of GFP- or mCherry-labeled DN neurons were transferred to Metamorph software (version 7.5; Molecular Devices). Maximum inscribed circles of cell bodies were drawn manually, and the diameter of the circles was regarded as the diameter of the cell. Labeled cells were categorized into 1) small-diameter (<12 µm) and 2) large-diameter (≧12 µm) neurons. This is because the diameter of most (>96%) inhibitory neurons as visualized by GFP signals in GAD67-GFP knock-in mouse [36] was <12 µm.

## Results

To investigate the potential in which cerebellar uRL/VZ progenitors can generate cerebellar neurons and glial cells, we introduced plasmids coding for fluorescent proteins by IUE, a method that allows introduction of plasmids into cells at the luminal surface of the cerebellar primordium. IUE was performed between E10.5 and E15.5, and the labeled cells were observed in the mature cerebellum.

### Labeled Cells in the Cerebellar Cortex

In preliminary experiments in which we utilized conventional plasmids, pCAGGS-mCherry (mCherry) or pCAGGS-EGFP (GFP), we observed the labeling of only a subset of cerebellar cells and none of stellate, basket or granule cells was labeled. Glial cells were not labeled either. Because cerebellar granule cell precursors undergo divisions in the EGL [5], [9]–[11], [37] and interneurons in the WM [12], [13], we suspected that repeated division of their progenitors compromised the labeling of cerebellar cell subsets. In an attempt to visualize cells that underwent repeated division, we electroporated Tol2-GFP plasmids into mouse embryos, which should allow for the integration of electroporated genes into the genome (reviewed in [22]). We also added mCherry to compare labeling by a conventional plasmid in the same animal. In most cases, cells labeled by conventional plasmids were examined after P21, an age when cell types can be easily identified by their morphological features.

#### 1) Neurons labeled by conventional plasmids

**Figure 1:**
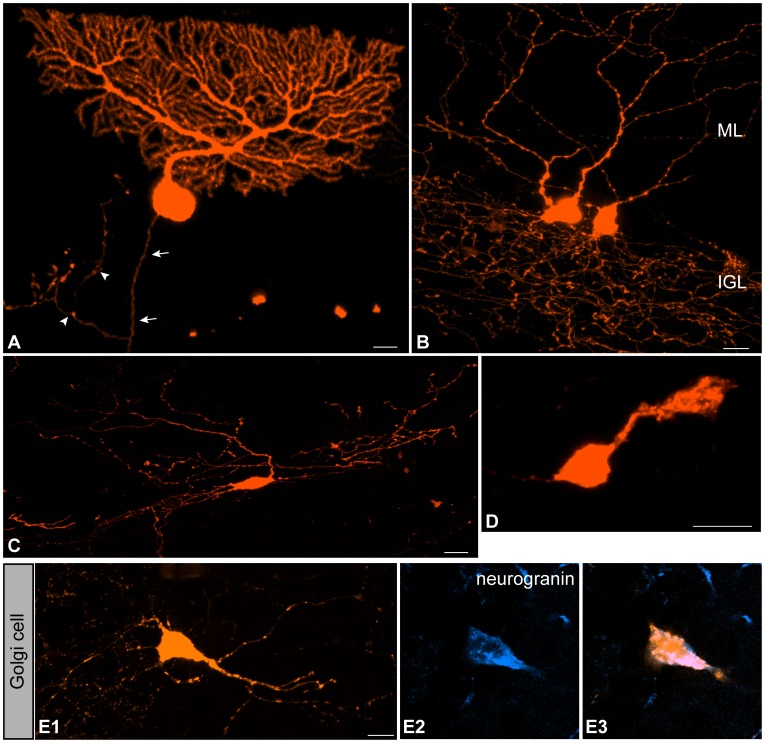
Neurons labeled in the cerebellar cortex by the electroporation of pCAGGS-mCherry. (A) A Purkinje cell. The cell extends an axon (arrows) towards the WM and axon collaterals (arrowheads) ending in the Purkinje cell layer. (B) Two Golgi cells in the IGL extending their dendrites into the ML and ramified axons into the IGL. (C) A Lugaro cell in the IGL, bipolar in shape, extending dendrites in parallel with the Purkinje cell layer. (D) A UBC in the IGL. (E1) An mCherry-labeled cell in the IGL shows the morphology of Golgi cells. Maximum projection images for a series of confocal images. (E2,E3) Double-labeling immunohistochemistry for neurogranin (blue) and mCherry (orange) confirmed the Golgi cell identity. Confocal plane images. ML, molecular layer; IGL, internal granular layer. Scale bars: A, 20 µm; B–E1, 10 µm.

Three types of neurons were labeled in the cerebellar cortex, Purkinje cells, Golgi cells, and Lugaro cells. These cells could be identified with typical laminar positions and morphologies. Purkinje cells, which formed a single-cell layer, extended a fan-like dendrite that spread in a narrow sagittal plane (Fig. 1A) and an axon towards the WM (Fig. 1A, arrows) [38]. Golgi cells, which are characterized by dendrites extending towards the molecular layer (ML) and an axon extensively ramifying in the internal granular layer (IGL) [38], were labeled beneath the Purkinje cell layer (Fig. 1B). These cells showed variation in size and position of their cell body (unpublished data). Identification of Golgi cells was supported by expression of neurogranin (Fig. 1E1–1E3) [39]. Lugaro cells were characterized by bipolar-shaped dendrites extending in parallel with the Purkinje cell layer and were observed in positions indistinguishable from those of Golgi cells (Fig. 1C) [40]. However, because it was sometimes difficult to distinguish Lugaro cells from Golgi cells on a morphological basis, we pooled them into a single category: Golgi/Lugaro cells. UBCs, typically characterized by a small cell body extending a single, short, and stubby dendrite, were also observed (Fig. 1D) [41]. These cells were localized at the IGL and mainly found inside the vestibulo-cerebellum (unpublished data), which is in agreement with previous studies [42], [43]. Thus, IUE of conventional plasmids caused the Golgi-like labeling of Purkinje, Golgi/Lugaro and UBC cells.

Labeling of these cells was developmental stage dependent. We found Purkinje cells were labeled in preparations electroporated from E10.5–E12.5, while the labeling of Golgi/Lugaro cells occurred by electroporation from E12.5–E15.5 (Table 1). UBCs were labeled only by electroporation at E13.5 and later (Table 1). These results indicate that the potential of the uRL/VZ progenitors responsible for generating these three cell types changes with development.

**Table 1:** Summary of cells in the cerebellar cortex labeled at each stage of electroporation.

Cell type	Constructs	E10.5	E11.5	E12.5	E13.5	E14.5	E15.5
***Inhibitory neurons***							
**Purkinje cell (PC)**	mCherry	++	++	++	−	−	−
	Tol2-GFP	++	++	++	−	−	−
	Cre*	++	++	++	NA	NA	NA
**Golgi/Lugaro cell (GC/LC)**	mCherry	−	−	+	+	+	+
	Tol2-GFP	+/−	+/−	+	+	+	+
	Cre*	+	+	+	NA	NA	NA
**stellate/basket cell (SC/BC)**	mCherry	−	−	−	−	−	−
	Tol2-GFP	++	++	++	++	++	++
	Cre*	++	++	++	NA	NA	NA
***Excitatory neurons***							
**unipolar brush cell (UBC)**	mCherry	−	−	−	+/−	+	+/−
	Tol2-GFP	+	+/−	+/−	+/−	+	+/−
	Cre*	−	+	+	NA	NA	NA
**granule cell (grC)**	mCherry	−	−	+/−	−	−	+/−
	Tol2-GFP	++	++	++	++	++	++
	Cre*	++	++	++	NA	NA	NA
***Glias***							
**astrocyte (As)**	mCherry	−	−	−	−	−	+
	Tol2-GFP	+	+	+	+	+	+
	Cre*	+	+	+	NA	NA	NA
**Oligodendrocyte (Ol)**	mCherry	−	−	−	−	−	+
	Tol2-GFP	+	+	+	+	+	+
	Cre*	+	+	+	NA	NA	NA
**Bergmann glia (BG)**	mCherry	−	−	−	−	−	−
	Tol2-GFP	++	++	++	++	++	++
	Cre*	++	++	++	NA	NA	NA

All sagittally sectioned samples in which both VZ- and uRL-derived cells were labeled were analyzed. Coronally sectioned samples were excluded from this table because it was difficult to systematically identify the labeled cells from their morphology in the coronal planes. mCherry, pCAGGS-mCherry; Tol2-GFP, a mixture of pCAGGS-T2TP and pT2K-CAGGS-EGFP; Cre*, pCAGGS-Cre was introduced into Ai9 mouse embryos. Number of samples from Tol2-GFP-introduced mice is three for each stage. Number of samples from Cre introduced Ai9 mice is as follows: n = 1 (electroporated at E10.5), n = 2 (electroporated at E11.5), n = 3 (electroporated at E12.5). +, ++: more than 4 cells were labeled in all samples; −: no cells were labeled in all samples; +/−: more than 4 cells were labeled in a part of the samples. NA: not available.

#### 2) Neurons labeled by Tol2-GFP

**Figure 2:**
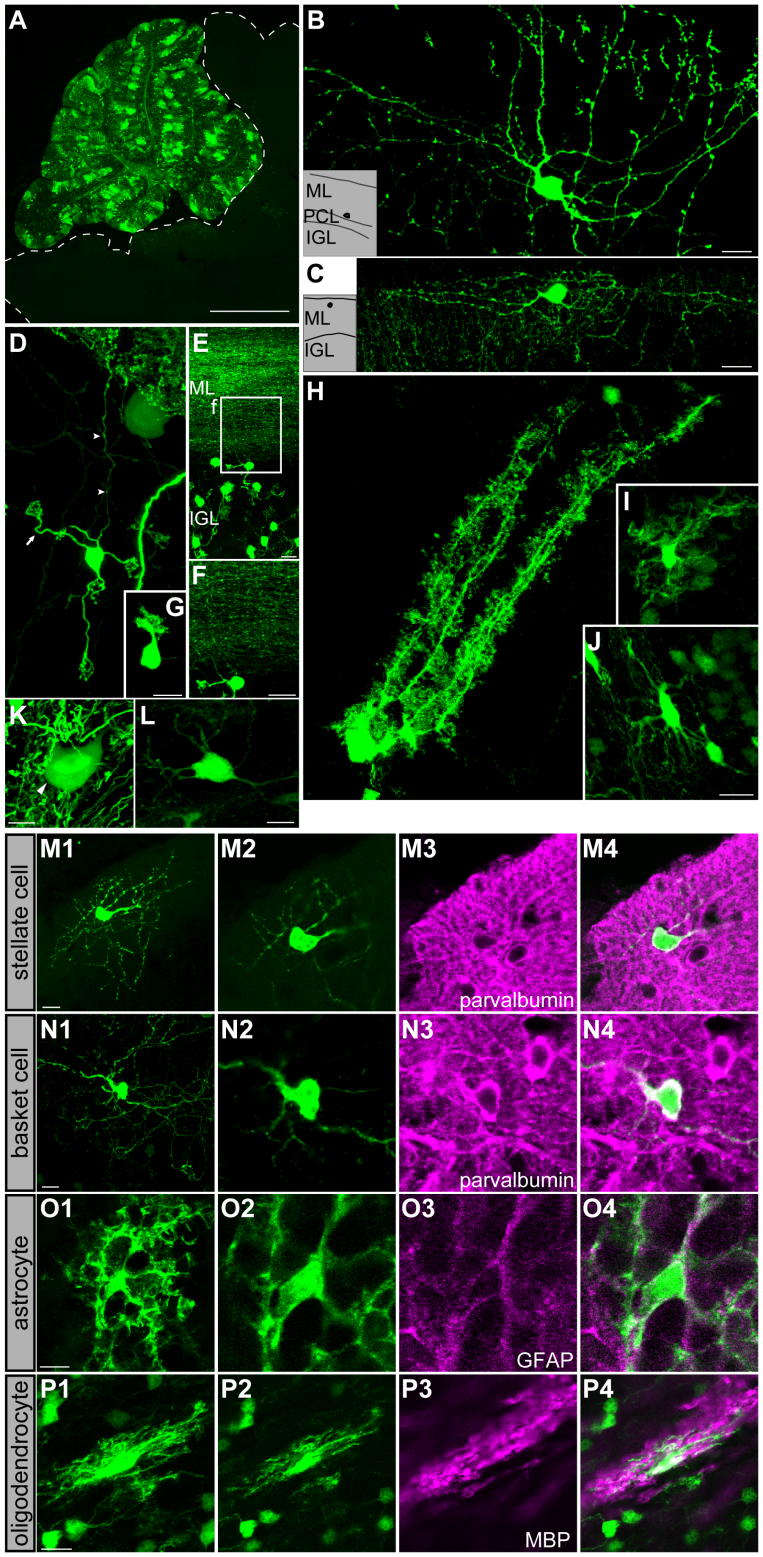
Labeled cells in the cerebellar cortex by IUE of transposon plasmids. (A) Low magnification view of a sagittal section of a Tol2-GFP electroporated cerebellum. (B) A basket cell extending its axon branches towards the Purkinje cell layer (lower left). (C) A stellate cell in the upper ML extending ramified processes. Black dots in the insets in B and C show the location of the respective cell bodies. (D) A granule cell with radiating dendrites (arrow) and an axon extending towards the ML (arrowheads). (E,F) Numerous parallel fibers occupy the ML. F is a high-magnification view of the boxed region in E. (G) A UBC in the IGL. (H) A Bergmann glia. (I) An astrocyte in the IGL. (J) An oligodendrocyte in the WM. (K) A large-diameter DN neuron (arrowhead). (L) A small-diameter DN neuron. (M1, N1, O1, P1) Maximum projection images for each series of confocal images of each cell. (M2–M4, N2–N4, O2–O4, P2–P4) Confocal plane images. (M1–M4) A Tol2-GFP labeled cell in the upper part of the ML shows the morphology of stellate cells. Double-labeling immunohistochemistry for parvalbumin (magenta) and GFP (green) confirmed the stellate cell identity. (N1–N4) A Tol2-GFP labeled cell in the lower part of the ML shows the morphology of basket cells. Double-labeling immunohistochemistry for parvalbumin (magenta) and GFP (green) confirmed the basket cell identity. (O1–O4) A Tol2-GFP labeled cell in the IGL shows the morphology of astrocytes. Double-labeling immunohistochemistry for GFAP (magenta) and GFP (green) confirmed the astrocyte identity. (P1–P4) A Tol2-GFP labeled cell in the white matter shows the morphology of oligodendrocytes. Double-labeling immunohistochemistry for MBP (magenta) and GFP (green) confirmed the oligodendrocyte identity. Scale bars: 10 µm ML, molecular layer; PCL, Purkinje cell layer; IGL, internal granular layer. Scale bars: A, 1 mm; B, C, E, F, K, L, M1, N1, O1, and P1, 10 µm; in G, 10 µm for D and G; in J, 10 µm for H, I, and J.

Because the dilution of plasmids can compromise the effectiveness of the labeling, we also used Tol2-GFP [22]. Under these conditions, it was expected that Tol2-GFP would label the cerebellar cells that could not be labeled by conventional plasmids. As expected, all cerebellar neuron types were labeled (Fig. 2) and the labeling of most cell types occurred after IUE at all developmental stages examined (Fig. 2 and Table 1).

Figures 2B and 2C show examples of a basket and a stellate cell, respectively, labeled by Tol2-GFP IUE at E10.5. These cells can be identified by small cell somata and ramified processes in the ML [38]. Basket cells were further characterized by their position in the lower part of the ML and axons that extended close to Purkinje cells (Fig. 2B, inset), while stellate cells tended to be localized in the upper ML and extended axons near the ML surface (Fig. 2C, inset) [38]. Identification of stellate and basket cells were further confirmed by immunostaining for parvalbumin (Fig. 2M1–2N4).

In most samples, numerous granule cells were also labeled by electroporation of Tol2-GFP. These cells were readily identified by their small and globular cell bodies and three or four short and radiating dendrites (Fig. 2D, arrow) [38]. In addition, granule cells extended their axons towards the ML perpendicular to the pial surface to form parallel fibers (Fig. 2D, arrowheads). Thus, in preparations that had many labeled granule cells, the ML was occupied by numerous parallel fibers (Fig. 2E,F).

Purkinje cell and UBCs were also labeled, but there was no difference in the labeling of Purkinje cells between the two labeling methods (Table 1 and unpublished data). UBCs were labeled by electroporation of Tol2-GFP at E10.5–E12.5 (Fig. 2G and Table 1), a period in which they were not labeled by mCherry.

Taken together Tol2-GFP caused cell labeling even where conventional methods failed and labeled all types of cerebellar cortical neurons.

#### 3) Labeling of glial cells

Tol2-GFP also labeled glial cells, which were rarely labeled when using mCherry. Labeling of all types of glial cells occurred irrespective of the stage of electroporation (Table 1). Fig. 2H shows an example of Bergmann glia, which can be identified by their distinct characteristic morphology and location [38]. Their cell bodies were located near the Purkinje cell layer and bore rugged, irregular, and expanded appendages. These cells extended two or more processes reaching the pial surface exhibiting leaf-like appendages. Astrocytes were also labeled: those in the WM were characterized by radially projecting processes with thin branches, while velate astrocytes observed in the IGL extended many processes and branches that were thicker, shorter, and more extensive than those in the WM (Fig. 2I). Both of them were stained for glial fibrillary acidic protein (Fig. 2O1–2O4). We also could identify oligodendrocytes, especially those in the WM, from their morphological features and expressions of myelin basic protein (Fig. 2P1–2P4). These cells had elongated, scrolled, and velamentous processes that extended along the axons in the WM (Fig. 2J). Collectively, these findings indicate that all types of glial cells in the cerebellar cortex originate from uRL/VZ progenitors at all developmental stages. The labeling of glial cells by Tol2-GFP, but not by conventional plasmids suggests that the progenitors for cerebellar glial cells undergo repeated division during embryonic stages (see below for exception).

### Progenitor Gene Recombination by Cre Recombinase also Labeled All Cell Types

**Figure 3:**
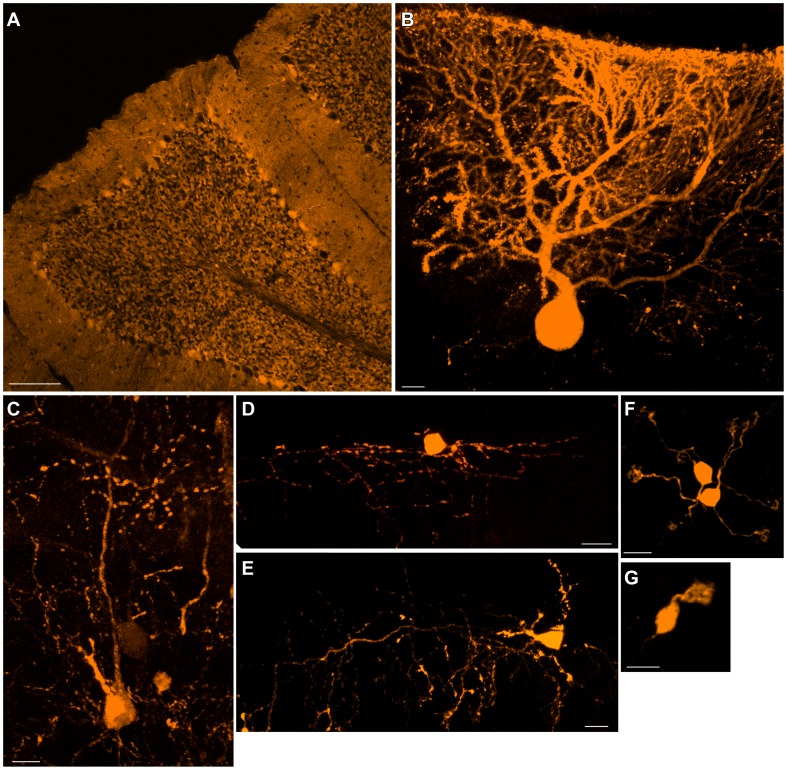
Electroporation of Cre recombinase into Ai9 reporter mice caused labeling of all cerebellar cell types. Electroporation was performed at E10.5–E12.5, and electroporated animals were observed at P21–P25 (see also Table 1). As shown in A, numerous cells were labeled in the cerebellum and included Purkinje cells (B), Golgi/Lugaro cells (C), stellate cells (D) and basket cells (E), granule cells (F) and UBCs (G), all of which were readily identified by their morphology and laminar positions. Glial cells, including Bergmann glia, were also labeled (data not shown). These findings support the hypothesis that the labeling of all cerebellar cell types by Tol2 transposon-mediated gene transfer was due to the integration of the GFP gene into the genome of the progenitors and the corresponding progeny. In cases in which many granule cells were labeled in the internal granular layer, it was difficult to identify Golgi/Lugaro cells based on morphological features only. In such cases, identification was facilitated by immunohistochemistry for neurogranin. Scale bar: A, 100 µm; B–G, 10 µm.

In principle, the difference in labeling between conventional plasmids and Tol2-GFP can be explained by whether the plasmid was integrated into the genome or not. This theory was confirmed by a third series of experiments in which we introduced Cre-recombinase expression plasmids to a reporter line with strong, ubiquitous expression of fluorescent proteins [23] (Fig. 3, Table 1). This method of labeling causes irreversible expression of tdTomato gene in electroporated progenitors. We obtained results identical to those obtained by Tol2-GFP (Fig. 3, Table 1), supporting our interpretation.

### Labeling of DN Cells

IUE also caused labeled cells in the DN (Fig. 2K,L) and the labeling was caused by both types of plasmids. DN neurons can be categorized into small-diameter inhibitory neurons and large-diameter excitatory neurons [44], [45]. The former should include inhibitory interneurons and nucleo-olivary neurons and the latter include nucleofugal excitatory neurons [46], [47]. We found both small- and large-diameter cells in the DN were labeled. Large-diameter (soma diameter ≧ 12 µm) cells were only labeled by electroporation at E12.5 and earlier (Fig. 2K, arrowhead) by both types of plasmids (Table 2). By contrast, small-diameter DN neurons (soma diameter <12 µm) were labeled at all stages (Fig. 2L) regardless of the plasmid type (Table 2). These findings suggest that small- and large-diameter DN neurons may emerge through distinct developmental mechanisms.

**Table 2:** Summary of cells in the DN labeled at each stage of electroporation.

Cell type	Constructs	E10.5	E11.5	E12.5	E13.5	E14.5	E15.5
**small DN (sDN)**	mCherry	+	+	+	+	+	+
	Tol2-GFP	+	+	+	+	+	+
	Cre*	+	+	+	NA	NA	NA
**large DN (lDN)**	mCherry	+	+/−	+/−	−	−	−
	Tol2-GFP	+	+/−	+	−	−	−
	Cre*	+	+	+/−	NA	NA	NA
**astrocytes in DN (As in DN)**	mCherry	−	−	+/−	+	+/−	+
	Tol2-GFP	+	+/−	+/−	+	+/−	+
	Cre*	+	+	+	NA	NA	NA

All sagittally sectioned samples in which both VZ- and uRL-derived cells were labeled were analyzed. Coronally sectioned samples were excluded from this table because it was difficult to systematically identify the labeled cells from their morphology in the coronal planes. mCherry, pCAGGS-mCherry; Tol2-GFP, a mixture of pCAGGS-T2TP and pT2K-CAGGS-EGFP; Cre*, pCAGGS-Cre was introduced into Ai9 mouse embryos. Number of samples from Tol2-GFP-introduced mice is three for each stage of electroporation. Number of samples from Cre introduced Ai9 mice is as follows: n = 1 (electroporated at E10.5), n = 2 (electroporated at E11.5), n = 3 (electroporated at E12.5). +, ++: more than 4 cells were labeled in all samples; −: no cells were labeled in all samples; +/−: more than 4 cells were labeled in subsets of all samples. NA: not available.

We also found astrocytes in the DN were labeled. Curiously, DN astrocytes were labeled by IUE of conventional plasmids even at E12.5 (Table 2) unlike cortical glial cells, suggesting distinct developmental mechanisms than these other glial cells.

### Cases of Labeled Cerebellar Cell Subsets

A technical limitation of IUE is that exclusive labeling of either the uRL or the VZ is usually difficult. Thus, in most cases, it could not be determined from which of these two regions the labeled cells derived (Table 1). However, in two cases only a subset of cerebellar cells were labeled even by permanent labeling methods, allowing their spatial origin to be investigated retrospectively. In the case where Cre-recombinase expression plasmids were electroporated into an E11.5 Ai9 embryo, no cells were labeled in the ML (Fig. S1A). Labeled cells consisted only of excitatory neurons, namely granule cells (Fig. S1B), large-diameter DN neurons (Fig. S1C), and UBCs (Fig. S1D). In the case where Tol2-GFP was electroporated to an E15.5 embryo, only stellate/basket cells, Golgi/Lugaro cells, Bergmann glias, oligodendrocytes in the WM and astrocytes in the IGL and DN were labeled (unpublished data). These results suggest that the three types of excitatory neurons have distinct origin from inhibitory neurons and glial cells. Given that all glial cells were labeled under the condition in which VZ-derived cells were specifically labeled, our results suggest that cerebellar glial cells including Bergmann glia originate from the VZ of the cerebellar anlage.

### Labeled Cells at the Site of Electroporation

**Figure 4:**
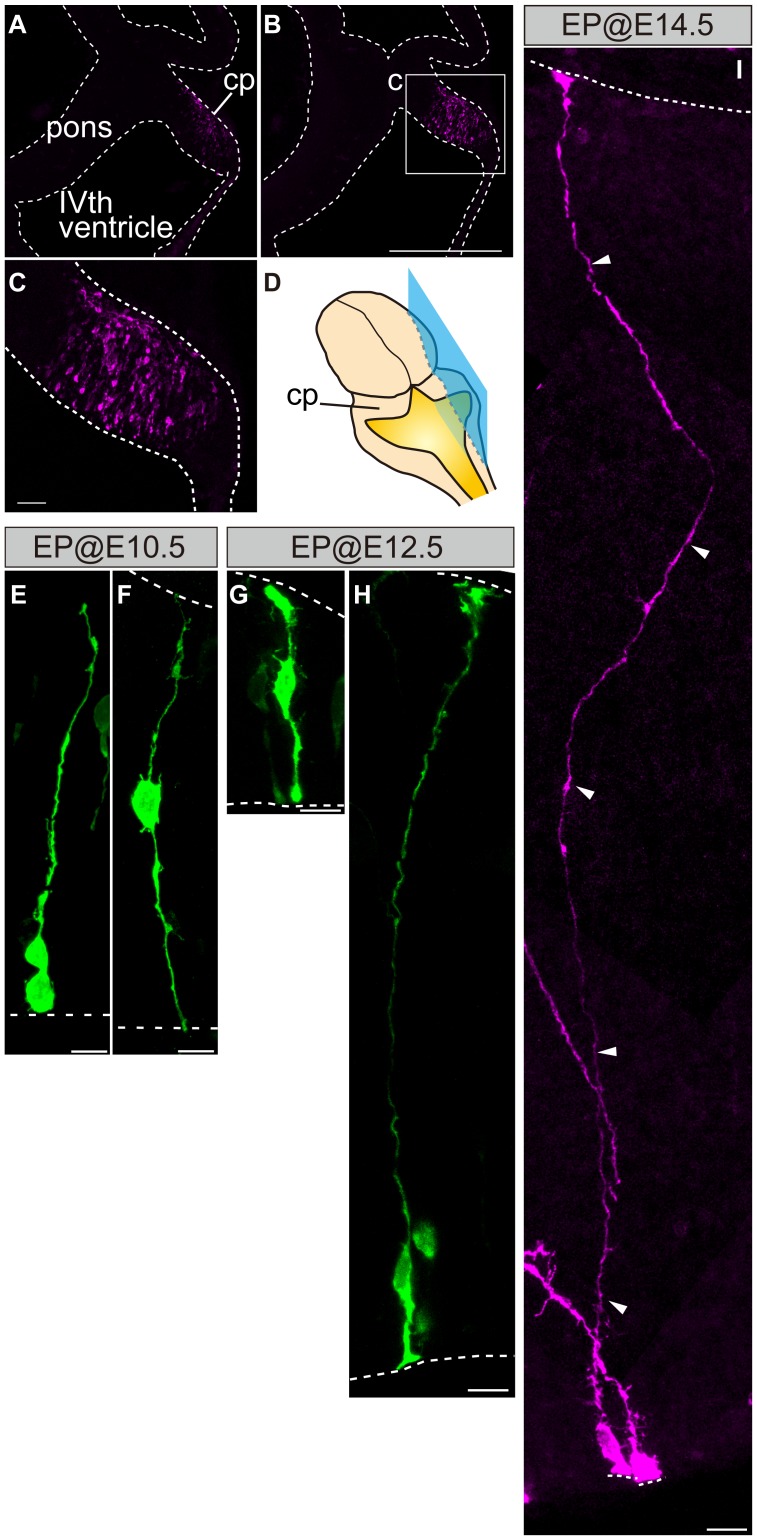
Labeled cells at the site of electroporation. (A,B) Labeled cells in the cerebellar primordium (cp). Electroporation at E10.5. (C) Magnified view of the boxed region (c) in B. (D) Schematic illustrating of dorsal view of the midbrain-hindbrain showing the plane of sections (blue). (E,F) Labeled cells attached to the ventricular surface directly (E) or by extending an apical process (F). (G,H) A cell with an apical endfoot and a radial process reaching the pial surface in the uRL region (G) and a cell with a radial glia-like morphology in the VZ (H). Electroporation at E12.5. (I) A radial glia-like cells in the VZ with a basal process (arrowheads) and apical process that reach the pial and ventricular surface, respectively. Electroporation at E14.5. (J) A mixture of pCAGGS-mCherry, pCAGGS-Cre, and pCALNL5-EGFP were electroporated and observed 12 h after electroporation. Parasagittal sections. cp, cerebellar primordium; VZ, ventricular zone. Scale bars: in B, 500 µm for A,B; C, 50 µm; E–J, 10 µm.

To examine the site of electroporation, embryos electroporated with a mixture of plasmids (mCherry, Cre, and pCALNL5-EGFP) were fixed for 12 h after electroporation at various times (E10.5, E12.5, and E14.5) and had their labeled cells in the cerebellar anlage observed (Fig. 4). For a majority of cases, labeled cells were found only within (Fig. 4A–D), and on one side (data not shown) of the cerebellar anlage (n = 8/11), although the lower rhombic lip or the brainstem was also faintly stained in the remainder of cases (n = 1 and 2, respectively) (unpublished data).

We next examined the labeled cells at the site of electroporation to analyze the morphological features of uRL/VZ progenitors. E10.5 IUE caused many labeled cells near the luminal surface of the uRL/VZ (Fig. 4C). Many of these cells attached to the ventricular surface directly (Fig. 4E) or by extending an apical process (Fig. 4F). These cells also extended basal processes, some of which reached the pial surface (Fig. 4F), and had morphologies that resembled radial glia of the neocortex [48]–[50]. Similar results were obtained 12 h after E12.5 electroporation. At this stage we also observed cells having an apical endfoot and a radial process that reached the pial surface in the uRL region (Fig. 4G) as well as in the VZ (Fig. 4H). After E14.5 electroporation, we also found labeled cells that attached to the ventricular surface and extended basal processes that reached the pial surface (Fig. 4I). These cells showed immunoreactivities for nestin and proliferating cell nuclear antigen (PCNA), a marker for a stem cell and a proliferating cell, respectively (unpublished data), supporting their radial glia-like nature. Collectively, these findings suggest that labeled cells in this study originate from radial glia-like progenitors in the uRL/VZ of the cerebellar anlage.

### Emigration of Neurons from the Cerebellar Primordium

**Figure 5:**
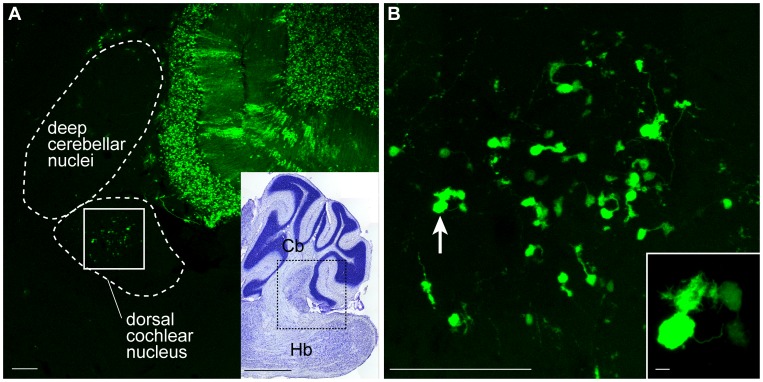
Emigration of neurons from the cerebellar primordium. UBCs labeled in the dorsal cochlear nucleus of the brainstem. (A) The fluorescent image is a higher magnified view of the dotted-boxed region in the inset, which is a Nissl-stained consecutive section. (B) A high magnified view of the boxed region (white solid line) in A. The inset is a magnified view of a UBC (arrow). Parasagittal sections. Cb, cerebellum; Hb, hindbrain. Scale bars: A, B, 100 µm; in the inset of A, 1 mm; in the inset of B, 10 µm.

The fact that we can specifically label progenitors in the cerebellar anlage allowed us to track cells that emigrated from the cerebellum. We found labeled cells in the dorsal cochlear nucleus in the samples in which no other labeled cells were observed in other regions of the brainstem (n = 8/18): electroporation at E10.5 and E11.5 caused labeled cells only in the Tol2-GFP introduced mouse or Cre introduced Ai9 mouse (n = 2 at each stage), while E14.5 and E15.5 electroporation caused labeled cells irrespective of the type of construct (n = 2 each). The morphology of these cells clearly indicated that they are UBCs (Fig. 5A,B). These findings indicate that UBCs in the dorsal cochlear nucleus have cerebellar origin.

## Discussion

Despite the abundance of studies on cerebellar cell development, the temporal relationship between uRL/VZ progenitors in the cerebellar primordium and their final phenotype still remains obscure. In the present study we used IUE to temporally or permanently label uRL/VZ progenitors at varying developmental stages and observed labeled cells in mature cerebellum. We here demonstrate that all types of cerebellar neurons and glias originate from cerebellar anlage, possibly from radial glia-like cells. Moreover, we show that the potential of uRL/VZ progenitors of respective cerebellar cell types to generate specific cerebellar cells or their precursors changes in a developmental-stage dependent manner, although some of them continue self-renewal up to E15.5.

### Radial Glia-like Morphology of Labeled uRL/VZ Cells

Examination of uRL/VZ cells labeled 12 h after electroporation revealed that a part of these cells display endfeet directly contacting the ventricular surface and basal processes reaching the pial surface, suggesting that uRL/VZ progenitors for cerebellar cells exhibit morphologies typical of radial glia, which are known as neuronal progenitors in the neocortex (reviewed in [49], [50]). That radial glia-like morphologies were observed up to E14.5 (Fig. 4) is consistent with the interpretation that uRL/VZ cells labeled by our method retain multipotency for a protracted period of time (see below). That nestin-positive radial fibers and PCNA signals were observed in the uRL/VZ even at E15.5 reinforces this interpretation.

### IUE as a Tool to Study the Development of Cerebellar Cells

In principle, it is not possible to control the site of labeled progenitor by our current method. However, in the present study, all cells labeled in the cerebellum likely have cerebellar origin for the following reasons. 1) When observed 12 h after IUE, labeled cells were confined to the cerebellar primordium in the majority of cases (Fig. 4). 2) All cell types were reproducibly labeled whenever the cerebellum was exclusively labeled (see Table 1). 3) When plasmids were accidentally introduced into only the midbrain or lower rhombic lip, no cerebellar cells were labeled (unpublished data).

The spatial origin of cerebellar cells has been extensively studied using GFM methods [8], [14], [15], [17]–[21], [51]. However, IUE has several advantages that can overcome some limitations of GFM. By varying the stage of electroporation, one can systematically study the developmental stage-dependent generation of cells from uRL/VL progenitors. Although developmental stage-dependent events can also be studied using inducible GFM [8], [14], the gene expression patterns usually change with development and the expressions are not necessarily confined to uRL/VZ progenitors [14], [15], [19]–[21]. Another point is that whereas GFM sometimes labels only a subset VZ cells (e.g., [8]), cells facing the luminal surface appear to be non-specifically labeled by IUE [25]. Finally, the whole cell, including axons and dendrites, can be labeled in a Golgi-like manner, enabling morphological identification of cells as well as tracking of axonal projections.

Although IUE non-specifically labels progenitors, it can be used to label specific types of cells in the cerebellum by choosing the stage of electroporation. For example, we could specifically label Purkinje cells by IUE of conventional plasmid at E11.5 and earlier (Table 1). Likewise, if an appropriate promoter/enhancer (for example, Ptf1a enhancer) is utilized for IUE, Golgi/Lugaro cells can be specifically labeled by IUE at E13.5 and later (Table 1) in the IGL.

Since IUE in principle allows introduction of any types of genes, we should be able to use this to manipulate functions of specific genes. By choosing stages of IUE, it is possible to manipulate specific genes in specific types of cerebellar cell by co-electroporating genes of specific function or shRNA.

### Time of Origin of Cerebellar Neurons

#### 1) Excitatory neurons

**Figure 6:**
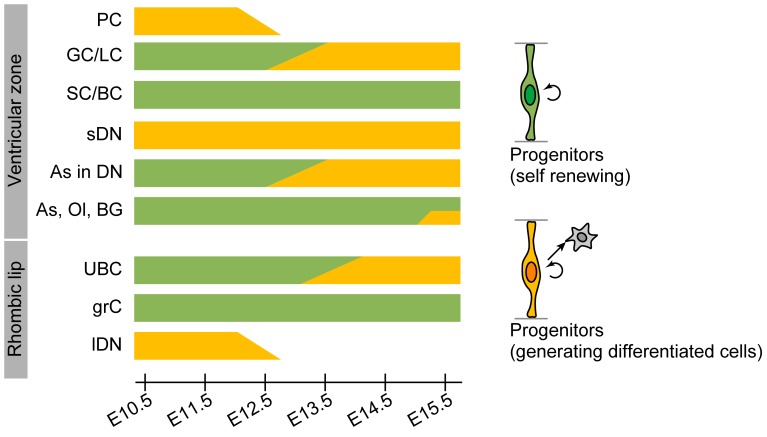
Schematic summarizing the present results. Progenitors having a radial glia-like morphology are present both in the VZ and uRL. Green bars signify that progenitors are self-renewing at indicated stages. Orange bars show stages at which progenitors are generating differentiated cells. GC/LC, Golgi cell and Lugaro cell; PC, Purkinje cell; SC/BC, stellate and basket cell; BG, Bergmann glia; As, astrocyte; Ol, oligodendrocyte; sDN, small-diameter deep nuclei neuron; lDN, large-diameter deep nuclei neuron; grC, granule cell.

Previous studies have suggested that granule cell precursors that originate from the uRL tangentially migrate to form the EGL, where they generate granule cells 2–3 weeks postnatally in the rat [3], [11] and these cells eventually migrate into the IGL [52]. Our results suggest that the uRL progenitors facing the IVth ventricle lumen are present at E10.5–E15.5 and continue to produce granule cell precursors. Moreover, the fact that granule cells were rarely labeled by mCherry even at E15.5 indicates that their uRL progenitors or their progeny continue proliferation at least up to this late stage (Fig. 6, grC).

UBCs, which are also thought to derive from the uRL [18], [43], were labeled by mCherry at E13.5 and later, but only by Tol2-GFP or Cre recombinase before then. These present findings suggest that uRL progenitors for UBCs are already present even at E10.5 (Fig. 6, UBC), while production of UBC cells is initiated only at E13.5. Results of BrdU labeling experiments, which demonstrated that these cells were generated only from E13.5 in rat (E11.5 in mouse) [53], are largely consistent with our findings.

Large-diameter DN neurons, which originate from the uRL [14], [15], were labeled only at E10.5–E12.5 (Fig. 6, lDN) and their labeling occurred regardless of the plasmid type (Table 2). Our findings indicate that the terminal differentiation of large-diameter DN neurons occurs almost directly from progenitors at E10.5–E12.5, which is largely consistent with the results of birthdating experiments (E13–E15 in rat [54] and E11–E13 [5] or E10–E12 [55] in mouse). Our finding that UBCs begin to be labeled around the stage when large-diameter DN neurons are not labeled any more (E13.5)(Fig. 6) raises the possibility that progenitors of large-diameter DN neurons in the uRL may begin to produce UBCs at later developmental stages.

#### 2) Inhibitory neurons

Purkinje cells, which are thought to originate from the VZ [8], [19], were labeled only at E10.5–E12.5 regardless of the plasmid type. This suggests that the VZ progenitors lose their potential to generate Purkinje cells or disappear with development (Fig. 6, PC). Moreover, the fact that Purkinje cells can be labeled by mCherry at E10.5-E12.5 suggests that the labeled progenitors almost directly produce Purkinje cells during this period, an interpretation supported by early tritium thymidine labeling experiments [5], [9].

Golgi/Lugaro cells were labeled by mCherry electroporation at E12.5 and later (Fig. 6, GC/LC), suggesting their progenitors produce these cells during this period. This result too agrees with the birthdating study by Miale and Sidman [5]. The fact that these cells begin to be labeled at a stage when Purkinje cells are not labeled any more (E13.5)(Fig. 6) raises the possibility that the progenitors that have generated Purkinje cells switch to produce Golgi/Lugaro cells, although Purkinje cells and Golgi/Lugaro cells might originate from distinct progenitors.

Stellate/basket cells are generated during 1–2 weeks postnatally [3], [6], [56], presumably in the WM [12], [57]. Our results showed that proliferating progenitors for these cells reside in the VZ at E10.5–E15.5 (Fig. 6, SC/BC). It remains unknown when the progenitors begin to leave from the VZ. In our preliminary experiments, in which IUE of GFP were performed at E13.5 and labeled cells were examined after BrdU administration one day later, many BrdU/GFP-double labeled cells were observed in the mantle layer of the cerebellar primordium. We also found that BrdU immunoreactivity was very low at the uRL/VZ at E15.5 (unpublished data). Taken together, these findings suggest that the progenitors for stellate/basket cells leave the VZ between E13.5 and E15.5, if not all.

### Time of Origin of Cerebellar Glial Cells

Our findings that the three types glial cells were labeled by the electroporation of Tol2-GFP or Cre recombinase at E10.5–E15.5 suggests that glial cell progenitors are present at the VZ throughout embryonic development (Fig. 6, BG, As, Ol). Among the three types of glial cells, Bergmann glias appear to undergo terminal differentiation by P5 (Fig. S2). However, differentiation of oligodendrocytes and astrocytes in the cerebellar cortex appears to occur later, because no differentiated oligodendrocytes and astrocytes were observed at this stage (unpublished data).

It is noteworthy that DN astrocytes were labeled by mCherry at E12.5 onward unlike other glial cells (Fig. 6, As in DN). Such early differentiation of DN astrocytes contrasts with the late terminal differentiation of astrocytes in the neocortex where astrocytes differentiate after neurogenesis [58]. Further studies including birthdating experiments are required to confirm early differentiation of DN astrocytes.

We have found evidence suggesting that Bergmann glia, astrocytes in the cerebellar cortex as well as those in the WM and oligodendrocytes all originate from cerebellar progenitor pools (Fig. 6). While there is evidence for an extracerebellar origin of oligodendrocytes [59], [60], our results show that at least some have cerebellar origin. Curiously, two samples in which genes appeared to be exclusively introduced into the VZ or uRL suggest that all glial cell types have a VZ origin (Fig. 6, upper panel) [8].

### Emigration of UBCs

A previous immunohistochemical study using a Tbr2 antibody reported a stream of labeled cell towards the brainstem in P0.5 preparation [43], suggesting emigration of UBCs into the brainstem. The present results that labeled UBCs were found in the dorsal cochlear nucleus directly demonstrated emigration of UBCs from the cerebellar anlage. The fact that these cells exhibit a characteristic morphology even after emigrating from the cerebellum suggests cell autonomous mechanisms for their morphological differentiation.

Taken together, the present results demonstrate that while all cerebellar cells have cerebellar origins, the cerebellum can also supply extracerebellar cells.

## Supporting Information

Figure S1 **Labeling of cerebellar cell subsets.** (A–D) In one example in which Cre recombinase was electroporated into an E11.5 Ai9 embryo, only granule cells, large-diameter DN neurons and UBCs were labeled. (A) Low-magnification view of the sagittal section of an Ai9 mouse cerebellum in which Cre plasmids were introduced at E11.5. (B) A granule cell in the IGL. (C) A large-diameter DN neuron. (D) A UBC extending an axon (arrowheads) in the IGL. ML, molecular layer; IGL, internal granular layer. Scale bar: A, 100 µm; B–D, 10 µm.
(TIF)

Figure S2 **A Tol2-GFP-labeled Bergmann glia in P5 cerebellum after the electroporation at E12.5.** (A) A labeled Bergmann glia extending radial processes. (B) Double-labeling immunohistochemistry for GFAP (magenta) and GFP (green) confirmed the identity of Bergmann glia. Scale bar: in I, 10 µm for A and B.
(TIF)
