# Supplementary figures and images for: Development of Cerebellar Neurons and Glias Revealed by in Utero Electroporation: Golgi-Like Labeling of Cerebellar Neurons and Glias
Source: PLoS One. 2013 Jul 23;8(7):e70091. doi: 10.1371/journal.pone.0070091 (PMC3720936; doi:10.1371/journal.pone.0070091)

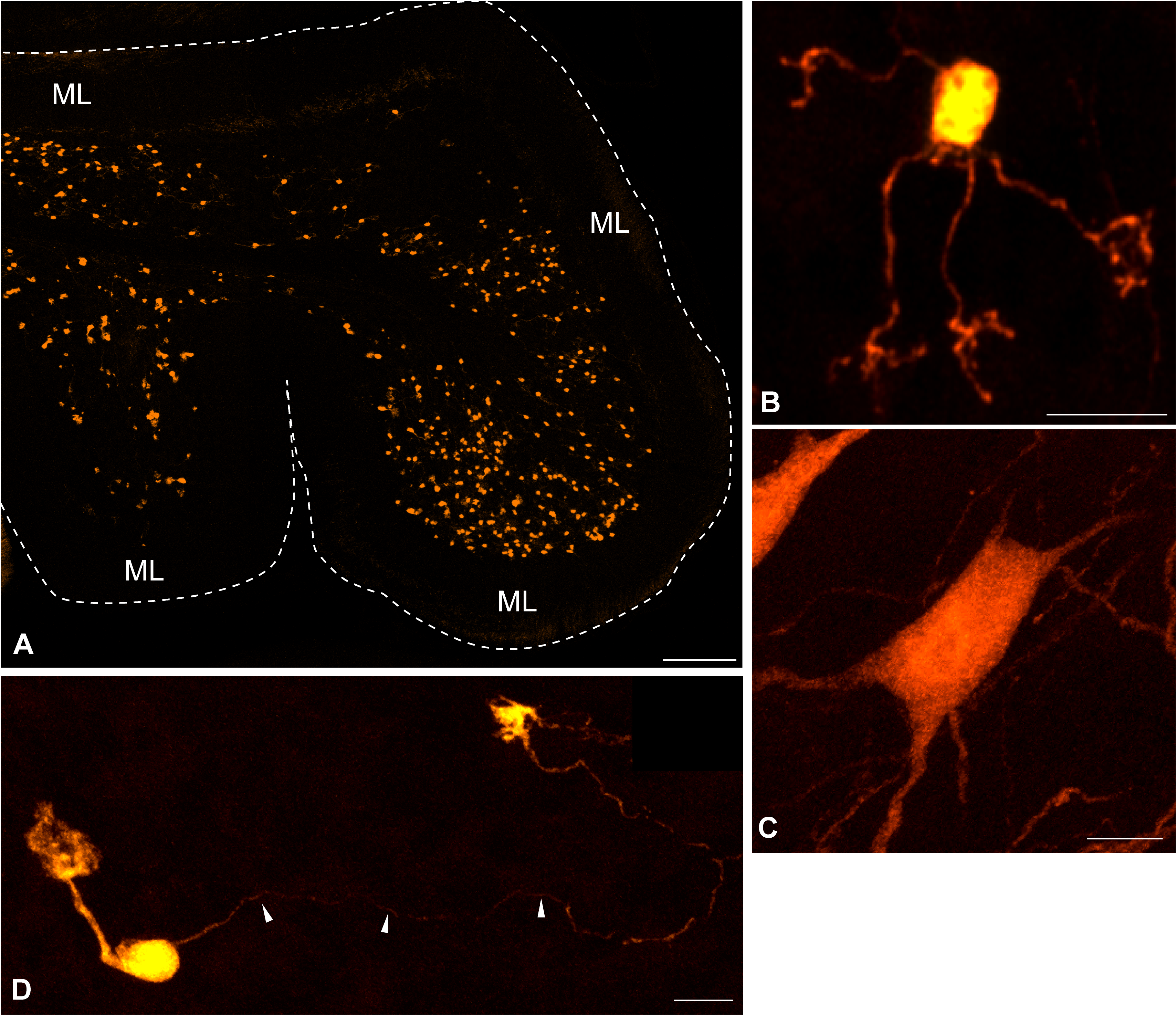

Supplement: Figure S1 — Labeling of cerebellar cell subsets. (A–D) In one example in which Cre recombinase was electroporated into an E11.5 Ai9 embryo, only granule cells, large-diameter DN neurons and UBCs were labeled. (A) Low-magnification view of the sagittal section of an Ai9 mouse cerebellum in which Cre plasmids were introduced at E11.5. (B) A granule cell in the IGL. (C) A large-diameter DN neuron. (D) A UBC extending an axon (arrowheads) in the IGL. ML, molecular layer; IGL, internal granular layer. Scale bar: A, 100 µm; B–D, 10 µm. (TIF) [file pone.0070091.s001.tif]

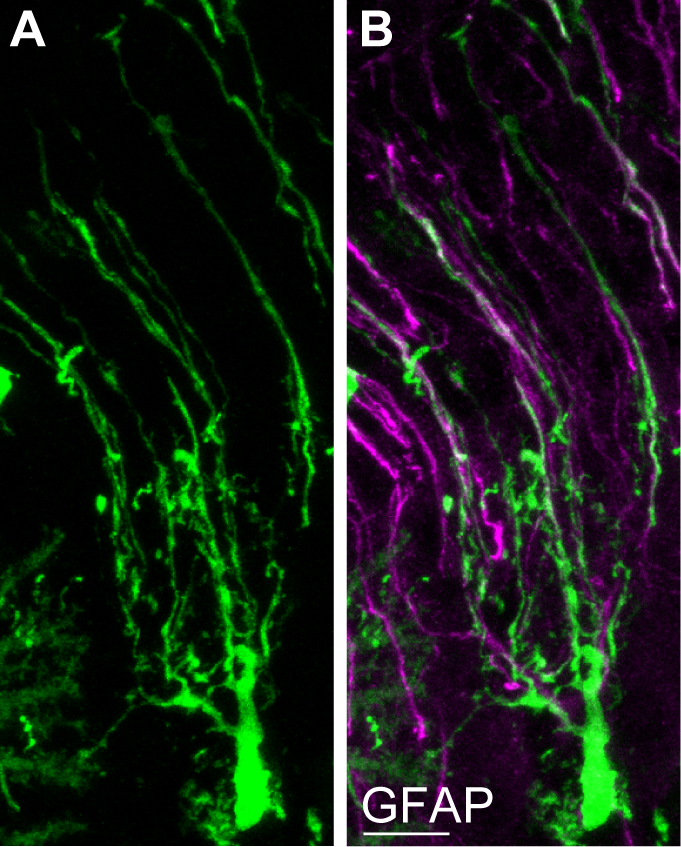

Supplement: Figure S2 — A Tol2-GFP-labeled Bergmann glia in P5 cerebellum after the electroporation at E12.5. (A) A labeled Bergmann glia extending radial processes. (B) Double-labeling immunohistochemistry for GFAP (magenta) and GFP (green) confirmed the identity of Bergmann glia. Scale bar: in I, 10 µm for A and B. (TIF) [file pone.0070091.s002.tif]
